# Left Atrial Appendage Management with the Watchman Device during Hybrid Ablation of Atrial Fibrillation

**DOI:** 10.1155/2019/4525084

**Published:** 2019-06-26

**Authors:** Mindy Vroomen, Bart Maesen, Justin G. Luermans, Harry J. Crijns, Jos G. Maessen, Mark La Meir, Laurent Pison

**Affiliations:** ^1^Department of Cardiology, Maastricht University Medical Center, Maastricht, Netherlands; ^2^Cardiovascular Research Institute Maastricht, Maastricht, Netherlands; ^3^Department of Cardiothoracic Surgery, Maastricht University Medical Center, Maastricht, Netherlands; ^4^Department of Cardiac Surgery, UZ Brussel, Brussels, Belgium

## Abstract

**Background:**

In the recent ESC/EACTS guidelines, left atrial appendage (LAA) occlusion or exclusion in patients undergoing (thoracoscopic) atrial fibrillation (AF) ablation surgery is recommended. The Watchman device (WD, Boston Scientific, Minnesota) has proved to reduce the risk of thromboembolic events by closing of the LAA, yet no data exist on WD implantation during surgical AF ablation. The objective is to determine if WD implantation is safe and feasible in a hybrid AF ablation setting (i.e., combination of thoracoscopic epicardial surgical and endocardial catheter ablation) and could become subject of further testing to serve as a bail-out in cases in which surgical LAA occlusion methods cannot be applied, due to, for example, severe adhesions.

**Methods:**

In this prospective, single center, pilot study, 10 consecutive patients undergoing a hybrid ablation qualifying for LAA exclusion (CHA_2_DS_2_-VASc ≥ 1) were included. At the end of the hybrid ablation, the LAA was occluded endocardially using the WD. The feasibility endpoint was successful implantation. The safety endpoint concerned major complications.

**Results:**

One patient was excluded and replaced because the LAA was insufficiently visible on transesophageal echocardiography. In 10/11 patients, device delivery was successful (mean time: 35 minutes). No major complications occurred. Transesophageal echocardiography after 6 weeks and 6 months showed successful occlusion of the LAA without significant peridevice flow.

**Conclusion:**

Implantation of the WD seems to be feasible and safe in the setting of hybrid AF ablation and could be an alternative to epicardial occlusion in surgical AF ablation procedures. Larger studies are required to confirm these findings. This trial is registered with NCT02471131.

## 1. Introduction

Thromboembolic stroke is one of the most feared complications of atrial fibrillation (AF). The risk of developing a stroke is increased 5-fold in patients with AF [1]. The left atrial appendage (LAA) is the origin of more than 90% of emboli in nonvalvular AF [2]. Antithrombotic therapy is effective, but its use can be contraindicated and is associated with risk of bleeding, suboptimal anticoagulation control, and poor compliance [3–5].

In the recent guidelines of the European Society of Cardiology (ESC) in collaboration with the European Association of Cardio-Thoracic Surgery (EACTS), surgical LAA occlusion or exclusion in patients undergoing (thoracoscopic) AF surgery is a Class 2b-B recommendation [6]. LAA occlusion as a stand-alone procedure in patients with contraindications for long-term antithrombotic treatment received the same classification. Several LAA occlusion techniques with variable success rates exist. Not only surgical occlusion with ligation or suture, but even exclusion with stapler showed high occurrence of unsuccessful closure [7, 8]. With a success rate of more than 98% of successful closure of the LAA, the AtriClip device (AtriCure Inc., Cincinnati, OH, USA) is an effective and safe method [9, 10]. However, in the rare case this device cannot be applied at the base of the LAA, for example in redo procedure with firm adhesions, the risk of stroke remains unaltered [11]. Another possibility to occlude the LAA is using the endocardial Watchman device (WD, Boston Scientific, St. Paul, MN, USA). This device has an implant procedure success rate of up to 98.5% and has proved to reduce the risk of thromboembolic events with acceptable procedure-related complication rates [12, 13], also when combined with catheter ablation for AF [14]. The potential downside of this endocardial device is that acute electrical isolation of the LAA is not obtained, which might be a consideration in the light of the LAA having a potential role as a trigger site of AF [15].

To the best of our knowledge, the WD has never been implanted during a surgical AF ablation. Since the WD has to be implanted under aspirin and heparin, this could possibly lead to an increase in bleeding risk for the surgical procedure. Therefore, this study aimed to determine if WD implantation in a hybrid AF ablation setting (i.e., combination of thoracoscopic epicardial surgical and endocardial catheter ablation) is safe and feasible and can become subject of further testing to serve as a bail-out in cases in which surgical LAA occlusion methods cannot be applied.

## 2. Methods

In this prospective nonrandomized, single center, pilot study, all consecutive patients scheduled for a hybrid AF ablation between August 2015 and October 2016 were screened. Patients are considered for a hybrid ablation if they fulfill at least one of the following criteria: (1) failure of at least one antiarrhythmic drugs (AAD) class I or III, (2) LA volume of ≥29ml/m2, (3) previous failed catheter ablation, (4) persistent or long-standing persistent AF, or (5) based on patient preference. As per protocol, 10 patients meeting the following inclusion criteria were included after informed consent for implanting the WD was obtained: at least 18 years of age; eligibility for a hybrid ablation; eligibility for at least short-term oral anticoagulation (OAC) therapy; absence of conditions requiring long-term OAC therapy, and CHA_2_DS_2_-VASc ≥ 1. Failure or impossibility of surgical LAA occlusion or expectation of being not suitable for surgical LAA occlusion was not an inclusion criterion for this study. All procedures were carried out at the Maastricht University Medical Center, Maastricht, the Netherlands, by the same surgeons (M.L.M. and B.M.) and electrophysiologist (L.P.). The usual learning curve and clinical experience with WD implantation were obtained by the implanting electrophysiologist before the present study. The study was approved by the local ethical committee and registered with ClinicalTrials.gov, number NCT02471131.

### 2.1. Hybrid Ablation Procedure

Two to three days before the ablation procedure, OAC was discontinued. The procedure was performed in a hybrid operating room under general anesthesia with double-lumen endotracheal tube placement for selective right lung ventilation. Transesophageal echocardiography (TEE) was used to confirm absence of LAA thrombi and to guide WD implantation. One 5 mm camera port and two 5 mm workings ports were inserted in the left hemithorax. The pericardium was opened posterior to the phrenic nerve. After blunt dissection of the pericardial reflections of the superior and inferior caval vein, antral isolation of the left and right pulmonary veins (PV), each side as a pair, was performed with a bipolar radiofrequency (RF) clamp (Synergy series, Atricure). Connecting lines between both superior PVs (roof line) and inferior PVs (inferior line) were made epicardially using a unilateral bipolar RF rail device (Coolrail, Atricure), creating a so-called box lesion.

Via a femoral venous approach, a His bundle (St. Jude Medical, St. Paul, MN, USA) and coronary sinus catheter (Medtronic, Minneapolis, MN, USA) were placed under fluoroscopy, followed by full heparinization using a dose of 1000E per 10 kg. After transseptal puncture, an activated clotting time >300 seconds was maintained. Patients who were still in AF after epicardial ablation were electrically cardioverted to restore sinus rhythm. The PVs and box lesion were mapped, and exit and entrance block were checked using a circular mapping catheter (Lasso, Biosense Webster, Diamond Bar, CA, USA). The endpoint for the ablation procedure was bidirectional block in each PV and in the box. In case of incomplete lesions, endocardial touch-up ablation was performed with a 3.5-mm cooled tip RF catheter (SmartTouch, Biosense Webster). If necessary, additional right and/or left atrial lesions were made at the electrophysiologist's discretion. At the end of the procedure, after the WD was implanted, half of the normally used dosage (2.5 mg) of protamine was intravenously administered to avoid thrombus formation on the WD. A chest tube was placed in the pleural cavity.

### 2.2. Watchman LAA Closure Procedure

According to the standard hospital protocol, the day before the procedure, 300 mg aspirin was administered intravenously. After completion of the epicardial and endocardial ablation, the WD was implanted in the same operating room as the ablation procedure. Heparin was not rebolused before the implantation. As previously described in detail [16], the WD is a self-expanding nitinol frame structure with fixation barbs and a permeable polyterephthalate membrane that covers the atrial surface. The available device sizes are 21, 24, 27, 30, and 33 mm. To have sufficient compression, the size of the device should be 10% to 20% larger than the maximum diameter of the LAA, measured in 4 TEE views (0°, 45°, 90°, and 135°). Via the preexisting transseptal puncture and with use of a 14-F access sheath and catheter-based delivery system, the WD was deployed at the ostium of the LAA. In case of suboptimal positioning, the device was retrieved and redeployed. The implantation was guided by fluoroscopy and TEE to verify the position, stability, and adequate device compression.

### 2.3. Follow-Up

Low-molecular-weight heparin in combination with aspirin 80 mg was started 6 hours after the procedure. On the second or third postoperative day, the same OAC regime as used by the patient before the procedure was restarted. In case of use of direct OAC, heparin was stopped. In case of use of coumarine derivatives, heparin was stopped when an adequate INR level was reached. Patients were treated with OAC and aspirin for at least 45 days. If the TEE after 45 days revealed complete closure of the LAA, or a residual peridevice flow ≤5 mm in width (measured using color Doppler), and no thrombus on the device, OAC was discontinued and replaced with clopidogrel 75 mg once daily. Six months after implantation, TEE was repeated. In case of complete closure of the LAA, or a peridevice flow ≤5 mm, clopidogrel was stopped and only aspirin continued indefinitely. In case of thrombus formation on the device or a peridevice flow >5 mm, anticoagulation was continued.

According to the standard hospital protocol, rhythm was determined at 3, 6, and 12 months after the ablation using at least an ECG, but preferably at least a 24-hour Holter examination. In case of symptoms, additional ECGs and/or Holter examinations were performed. Recurrence was defined, according to the guidelines, as any supraventricular arrhythmia (AF, atrial flutter and atrial tachycardia) lasting >30 seconds [17].

### 2.4. Endpoints

The Primary Safety Endpoint comprised, in accordance with previous WD publications [18, 19], major complications: death <7 days after the procedure, ischemic or hemorrhagic stroke, device embolization requiring retrieval, device related complications requiring open surgery or major endovascular intervention, any bleeding related to the device necessitating an interventional procedure or packed red blood cell transfusion ≥2 units within 24 hours, pericardial effusion requiring intervention <3 months after the procedure, and femoral arteriovenous fistula.

The Primary Feasibility Endpoint was device delivery success. This was defined as successful delivery and release of the WD into the LAA, including successful retrieval and redeployment if necessary, and a residual peridevice flow ≤5 mm in width.

### 2.5. Statistics

Data were prospectively entered into a database. Data were analyzed using SPSS 24.0 (SPSS Inc., Chicago, IL, USA). Continuous variables with normal distribution were presented as mean ± standard deviation (SD); nonnormal variables were reported as median and interquartile range [IR] and categorical variables as frequencies with percentages.

## 3. Results

### 3.1. Study Population

Of the 38 patients who have been screened, 19 patients met the inclusion criteria and were informed about this study. The main reason for exclusion was a CHA_2_DS_2_-VASc of 0. Other reasons were contraindication for OAC due to recurrent bleedings, reoperation, kidney dysfunction, and being unable to be present at follow-up. Eleven patients gave informed consent.

### 3.2. Patient Characteristics

The baseline characteristics are summarized in Table 1. The median time between diagnosis of AF and the procedure was 33 months [IR 21-94]. The median CHA_2_DS_2_-VASc was 2 [IR 2-5], with a minimum of 1 and a maximum of 5. All patients used OAC before the procedure. None of the patients experienced ischemic events. Five patients underwent a prior endocardial catheter ablation procedure, of which 4 were for AF.

### 3.3. Watchman Device Implantation

**Figure 1:**
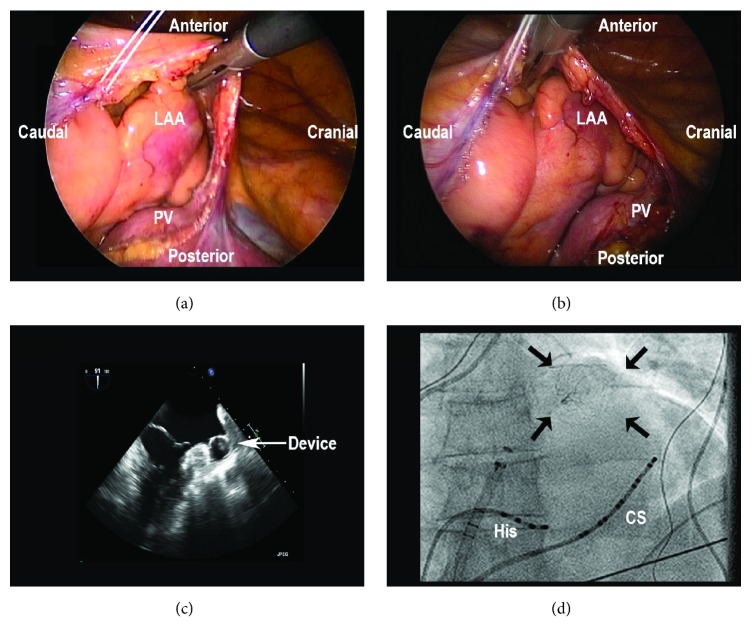
*Watchman device implantation - Intraoperative images*. (a) Left-sided thoracoscopic view of the left atrial appendage (LAA) before implantation. PV = Pulmonary vein. (b) Left-sided thoracoscopic view of the LAA after implantation, showing that implantation of the device does not lead to significant morphological changes of the LAA. (c) Per-procedural echocardiographic view after implantation. (d) Per-procedural thoracic radiographic image after implantation. His = His Bundle catheter, CS = Coronary Sinus catheter.

The Primary Feasibility Endpoint was met in 10/11 procedures. In three patients, the LAA consisted of a single lobe, in 6 patients of 2 lobes, and in one patient of multiple lobes. One patient had to be excluded before the implantation procedure was started because the LAA was insufficiently visible on TEE, so the measurements necessary for the implantation could not be performed. All other 10 devices could be delivered successfully and the residual peridevice flow was ≤5 mm in width in all patients at 45 days and 6 months after implantation [Figure 1]. Eight devices were deployed and released at first attempt, one device was retrieved twice, and in one patient a 27 mm device was changed for a 30 mm device because of incomplete occlusion of a side lobe. One other patient received a 30 mm device, while the other used sizes were 21 mm in 5 patients and 24 mm in 3 patients. The total procedure time was 4 hours and 28±49 minutes (range: 3h10m – 5h28m), which includes the device implantation time amounting 35±9 minutes (range: 3–50). The total radiation time was 11±4 minutes (range: 5–17), including the radiation time for WD implantation of 6±3 minutes (range: 2–11). The total radiation dosage was 53±25 mGy (range: 21–88), including that for the WD implantation of 31±7 mGy (range: 9–54). The amount of contrast which was used for the WD implantation was 84±39 mL (range: 30–165).

### 3.4. Complications

No major complications (as defined in previous WD publications [18, 19]) occurred, meaning the primary safety endpoint was met in all patients. Complications that occurred during or after the hybrid procedure but were not necessarily related to the device implantation of the device comprised 1 pneumothorax drained with a pleural catheter, 1 transient elevated right hemidiaphragm, 1 pericarditis, and 1 hospital admission because of tachycardiomyopathy with reduced left ventricular function of 25% due to recurrence of an atrial tachycardia. For one other adverse event, blood transfusion (≤2 units of packed red blood cells) two days after the procedure due to excessive blood loss through the thoracic drain on the operative day, a possible relation with the periprocedural anticoagulation necessary for the WD implantation cannot be excluded. After intravenous administration of protamine on the ward, the bleeding stopped.

### 3.5. Follow-Up

After 6 weeks none of the TEEs showed thrombus formation or migration of the device. The peridevice flow was ≤5 mm in all cases, with a mean of 1.2±1.5 [range 0-4]. OAC was stopped and clopidogrel started in all patients except one (tachycardiomyopathy).

At follow-up at 3 months, 5 patients received a 24-hour Holter and 3 patients a 7-day Holter. In 2 patients only ECGs were made. No recurrences were documented. Three patients were using AADs. In one patient, OAC was restarted due to side effects of clopidogrel.

After 6 months, none of the TEEs showed thrombus formation or device migration and the peridevice flow was ≤5 mm in all cases, with a mean of 1.1±1.4 [0-3]. Six patients showed complete closure of the LAA. In all patients a Holter was performed: 6 24-hour Holters, 1 48-hour Holter, and 2 7-day Holters; and in one patient a Reveal was implanted. OAC was restarted and aspirin stopped in a patient with hyperthyroidism and AF recurrences, for which a redo ablation procedure was considered necessitating the use of OAC. In all other patients, OAC or clopidogrel was stopped. While one other patient suffered recurrences of atrial tachycardia, the other 8 patients maintained sinus rhythm, of which 3 were with the use of AAD.

A 24-hour Holter after 12 months was performed in 4 patients, a 48-hour Holter in one, a 7-day Holter in 2. One patient was monitored via the reveal, in one patient a pacemaker was implanted, and in one patient only ECGs were made. Eight patients maintained sinus rhythm, all without the use of AAD. During the entire follow-up, no stroke occurred.

## 4. Discussion

This study is the first to show that it is feasible and safe to implant a WD during a hybrid AF ablation. The device was implanted successfully in all patients without major complications. TEE after 6 months' follow-up showed either persistent closure of the LAA or a peridevice flow of ≤5 mm.

In accordance with the ESC/EACTS guidelines [6], stand-alone endocardial closure of the LAA is mainly performed in patients who are ineligible for OAC due to (recurrent) bleedings, in patients who have a high bleeding risk due to comorbidities, and in patients who have suffered ischemic events despite adequate use of OAC. However, this recommendation is based on the PROTECT AF [18] and PREVAIL [19] studies. These randomized trials investigated the implantation of the WD, but patients with high risk for bleeding were not included. In patients undergoing (thoracoscopic) AF surgery, a surgical method of LAA exclusion is recommended to be considered [6, 20–22]. One advantage of surgical exclusion or occlusion is that electrical isolation of the LAA is obtained, which could favorably influence rhythm outcome [15, 23]. The success rate of this report seems comparable to previous publications using an AtriClip; however, due to the feasibility aim of the current study and the low patients' number, we cannot compare outcomes of WD with AtriClip or draw any well-founded conclusions [24, 25]. Further, in patients in whom the use of OAC is strictly contraindicated, the possible consideration to cease OAC after epicardial LAA occlusion becomes relevant, since for the implantation of the WD it is currently recommended to use dual antiplatelet therapy for at least 3 months and aspirin for at least 12 months. This is different from the strict OAC regime which was applied in this study, since drug regime changes were made in August 2017 based on study results: Sharma et al. [6] showed in 150 patients with a mean CHA_2_DS_2_-VASc of 4.4 that aspirin combined with clopidogrel in the first 6 months led to an ischemic stroke rate of 1.8 per 100 patient years after 5 years of follow-up. Further, a subanalysis of the EWOLUTION registry revealed an ischemic stroke risk at 1 year of 1.4% in patients using only dual antiplatelet therapy [26, 27]. The LAAOS III trial [28], exploring the efficacy of LAA occlusion for stroke prevention, and the ASAP-TOO trial [29], assessing the safety and efficacy of WD implantation in patients unsuitable for OAC, are currently including patients.

During surgical treatment of AF, it is common practice to exclude the LAA epicardially. Due to high occurrence of unsuccessful closure with ligation, (stapler) resection and suture closure, the AtriClip was developed. Its safety and efficacy, in occluding the LAA and reducing stroke, have been reported [9, 10]. Despite placing the device at the base of the LAA can be challenging in rare cases, it is possible to reach an implantation success rate of >98% with relative short implantation times [9, 10]. In case of incomplete closure the risk of thromboembolic complications can be increased [11], but a residual stump or pouch up to 1 cm is considered as successful occlusion. Although there might be an economic barrier in specific situations to deviate to the WD, this approach seems to be a considerable alternative in hybrid AF procedures without possibilities to place the clip at the base of the LAA, due to unsuitable anatomy or firm adhesions. The WD device is a frequently implanted endocardial LAA occluder. A meta-analysis [12], combining the data of the PROTECT AF, PREVAIL, and an additional registry [30], showed that all-cause stroke and systemic embolism rates per 100 patient years were similar for WD compared to OAC, respectively, 1.75 and 1.87 (p=0.94). Ischemic strokes, including the procedure-related strokes, occurred more in the WD group (1.6 vs. 0.9, p=0.05); however, haemorrhagic strokes were significantly less common (0.15 vs. 0.96, p=0.004) and there was a significant reduction in cardiovascular and unexplained death (1.1 vs. 2.3, p=0.006). In the current study no strokes occurred; however, only a small number of patients were included which prevents drawing conclusions.

In the PROTECT AF trial, safety, defined as 7-day procedure- and device-related complications, was a concern (8.7%). However, in the PREVAIL trial and EWOLUTION registry, the procedural safety had significantly improved (4.2% and 2.8%, respectively). Procedural and device-related strokes decreased from 1.1% in PROTECT AF to 0.4% in PREVAIL (p = 0.007) and 0.1% in EWOLUTION, while successful implantation increased from 88% in PROTECT AF to 98.5% in EWOLUTION. This is commonly seen in new interventional procedures and mainly depends on operator experience. The current result of 100% implantation success in this study is therefore explained by the experience of the implanting physician (L.P.). One of the other concerns about the WD is the acceptance of a Doppler peridevice flow up to 5 mm in width as a marker for successful implantation, because this might imply a source of thrombus formation. However, looking at published stroke rates applying this marker, this does not seem to be thrombogenic. A possible reason could be the small ostium which gives a high flow velocity and therefore less thrombogenic risk. Finally, an additional problem when implanting the WD during a hybrid AF ablation can occur. The bleeding risk might be increased since the surgical procedure is performed under aspirin, and the administered heparin for the endocardial part of the ablation is not fully antagonized at the end of the procedure. In our series, we report 1 patient needing transfusion, compared to <5% in procedures without WD implantation. Other complications are not increased compared to hybrid ablation using an AtriClip [24, 25].

### 4.1. Study Limitations

Due to the pilot nature of the study, only a limited number of patients were included. More data are needed to be able to draw definitive conclusions.

## 5. Conclusion

Implantation of the endocardial Watchman device seems to be feasible and safe in the setting of a hybrid AF ablation and could be an alternative to epicardial exclusion and occlusion methods in surgical AF ablation procedures. Larger studies are required to confirm these findings.

## Figures and Tables

**Table 1:** Patient characteristics.

General characteristics	No (%), Mean ± SD or Median [IR]
Male	5 (50%)

Age (years)	65 ± 4

Body Mass Index (kg/m^2^)	29.5 ± 6

CHA_2_DS_2_-VASc	2 [2-3]

AF characteristics	No (%) or Median [IR]

Paroxysmal AF	2 (20)

Persistent AF	7 (70)

Long-standing persistent AF	1 (10)

Atrial Flutter	3 (30)

AF duration (months)	33 [21-94]

Echocardiography	No (%) or Mean ± SD

Left ventricular ejection fraction (%)	56 ± 8

LA size (mm), LA volume (mL), LA volume index	47 ± 6, 99 ± 22, 50 ± 13

RA volume (mL)	75 ± 31

Moderate to severe valvular disease	0

SD = Standard Deviation, IR= interquartile range, AF = Atrial Fibrillation, OAC = Oral Anticoagulation, LA = Left Atrium, RA = right atrium.

## Data Availability

The data used to support the findings of this study are restricted by the ethical board MUMC/AZM in order to protect patient privacy.
